# Complete genome sequences of two *Exiguobacterium alkaliphilum* isolates from the Brazilian Pantanal hypersaline lakes

**DOI:** 10.1128/mra.00650-25

**Published:** 2025-10-24

**Authors:** William Lautert-Dutra, Francine Melise dos Santos, Amanda Pasinato Napp, Clarissa Lovato Melo

**Affiliations:** 1 Environmental Monitoring and Biotechnology Laboratory, Institute of Petroleum and Natural Resources (IPR), Pontifical Catholic University of Rio Grande do Sul (PUCRS) 28102, Porto Alegre, Rio Grande do Sul, Brazil; Indiana University, Bloomington, Bloomington, Indiana, USA

**Keywords:** hybrid genome assembly, bioremediation, environmental pollution, extreme environments, Halophilic

## Abstract

The climate crisis has stimulated research into alternative solutions for combating climate change and environmental pollution. Extremophilic microorganisms present a valuable opportunity to detoxify and degrade pollutants. We provide the complete genome of two *Exiguobacterium alkaliphilum* isolates from a hypersaline alkaline lake and emphasize their potential as bioremediation facilitators.

## ANNOUNCEMENT

At the Institute of Petroleum and Natural Resources (IPR), we sequenced two strains (BS282 and BS283) recovered from the biobank collection at the IPR-PUCRS to explore their biotechnological capabilities. The samples represent a hypersaline alkaline lake from sediment in the *Pantanal Nhecolândia* region of Brazil (19°30′49″ S, 56°10′01.8″ W). After removing the top layer of the sediment, the apical portion was plastic tubes at 4°C. Isolation used saline medium (10 g/L yeast extract, 100 g/L NaCl, 3 g/L C_6_H_5_Na_3_O_7_, 2 g/L KCl, 1 g/L MgSO_4_, 280 µg/L MnCl_2_, 0.05 g/L FeSO_4_, and 3 g/L NaHCO₃) (1, 2). Subcultures were developed to acquire final pure cultures maintained in a nutrient-rich medium (BHI). Each isolate was verified using Gram staining to discard contamination. For long-read sequencing, DNA extraction was performed with QIAcube (DNeasy Blood & Tissue, QIAGEN) following the manufacturer’s recommendations for bacterial DNA extraction. The Agilent TapeStation (Agilent Technologies) was used for DNA quality control. A Nanopore library (Oxford Nanopore Technologies) was constructed using the Ligation Sequencing gDNA Native Barcoding Kit 96 V14 (SQK-NBD114.96). Library sequencing was done using FLO-PRO114M: PromethION R10 (M Version) at Life Sciences Core Facility (LaCTAD; Campinas, Brazil). Basecalling was done using Dorado v 1.0.2 (r1041_e82_400bps_sup_v4.3.0). Short-read sequencing was conducted as previously described using lysis buffer (NeoSampleX) for lysis and magnetic beads for DNA extraction. Illumina NextSeq 1000 technology (2 × 300 bp, P1-600 Illumina kit) for sequencing (1).

**Fig 1:**
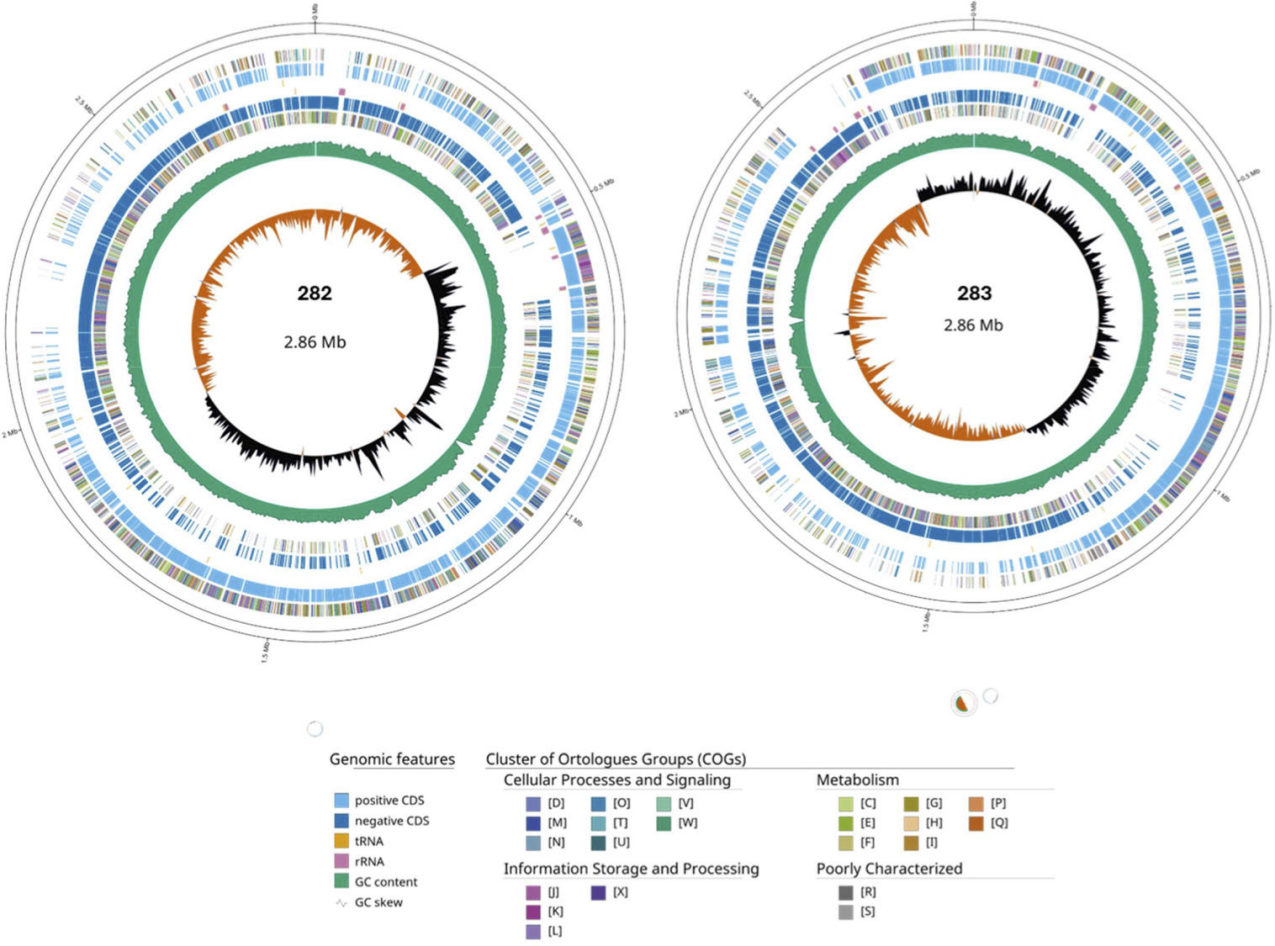
Complete *de novo* assembly for two *Exiguobacterium alkaliphilum* isolates. Overview of the genomes of isolates 282 and 283 derived from hypersaline lakes in Pantanal, Brazil. The genomes display COG functional categories mapped to the predicted metabolic coding regions in the assembled genomes. CDS, coding sequence; tRNA, transfer RNA; rRNA, ribosomal RNA; GC, guanine-cytosine; D, cell cycle control, division, chromosome partitioning; M, cell wall/membrane/envelope biogenesis; N, cell motility; O, post-translational modification, protein turnover, chaperones; T, signal transduction mechanism; U, intracellular trafficking, secretion, and vesicular transport; V, defense mechanism; W, extracellular structures; Y, nuclear structure: Z, cytoskeleton; A, RNA processing and modification; B, chromatin structure and dynamics; J, translation, ribosomal structure, and biogenesis; K, transcription; L, replication, recombination, and repair; X, mobilome: prophages, transposons; C, energy production and conversion; E, amino acid transport and metabolism; F, nucleotide transport and metabolism; G, carbohydrate transport and metabolism; H, coenzyme transport and metabolism; I, lipid transport and metabolism; P, inorganic ion transport and metabolism; Q, secondary metabolites biosynthesis, transport, and metabolism; R, general function prediction only; S, function unknown; and COG, Clusters of Orthologous Groups. Generated by GenoVi (https://github.com/robotoD/GenoVi).

PE short reads were filtered using *FastQC* v0.11.9 and *fastp* v0.23.4 (*trim-poly-g -detect-adapter minlength 90* bp) (3, 4). Long reads were trimmed using *Filtlong* v0.2.1, and short reads were used as k-mer matches (‘*PE short-reads’ +* ‘*--min_length 1000—keep_percent 90*’) (5). We used *Trycycler* v0.5.5, a consensus long-read assembler for genome assembly (6). We used *Flye* v2.9.5-b1801 (*--nano-hq*), *Minipolish* (*miniasm_and_minipolish.sh*) v0.1.2, and *Raven* v1.8.3 (*--disable-checkpoints + --graphical-fragment-assembly*) for assembly (7–9). Genomes were manually curated using *Bandage* v0.8.1, and incomplete assemblies were removed (10). The final consensus was polished using *Medaka* v2.0.1 (*-m r1041_e82_400bps_sup_v4.3.0*) and a short-read polishing using *Polypolish* v0.6.0 and *Pypolca* v0.3.1 (*—careful*) (11, 12). The presence of plasmids was evaluated using *Plassembler* v1.6.2 (13). We used the BUSCO tool v5.8.2 to assess the genome’s completeness (14). Annotation was done using *PROKKA* v1.14.6 and *PGAP* v2024-07-18.build7555 pipelines (15, 16). The complete genome assemblies and functional annotation of coding sequences are summarized in Figure 1, highlighting the distribution of COG functional categories across both isolates. The presence of metabolism linked to hydrocarbon degradation, plastic degradation, and biosurfactant production was determined using *HADEG* (*HADEG* v231119) (17). Assembly QC was evaluated with *QUAST* v5.2.0 (18). Default parameters were used, except where otherwise noted.

Illumina and Nanopore sequencing produced 14,239,938 and 714,692 (N50 8,730) reads for BS282, and 14,672,202 and 518,612 (N50 9,981) reads for BS283. Total trimmed sequences were 10,961,780 and 358,089 for BS282 and 11,832,006 and 274,027 for BS283. The average coverage for Illumina and Nanopore was 640× and 566×, respectively. For BS283, coverage depths reached 545× and 687×. General statistics for genome assembly and annotation are summarized in Table 1. The genomic insights from strains BS82 and BS283 highlight the potential of *E. alkaliphilum* in environmental biotechnology. HADEG analysis identified key genes involved in the aerobic degradation of alkanes, toluene, and PLA plastics.

**TABLE 1:** General statistics for the genome assembly and annotation of two *Exiguobacterium alkaliphilum* isolates obtained from hypersaline lakes from Brazilian Pantanal

Step	Features	282	283
Assembly (chromosome)	Genome size (bp)	2,860,491	2,860,492
	DNA G + C (%)	53.47	53.47
	DNA scaffolds	1	1
	N50	2,860,491	2,860,492
	N90	2,860,491	2,860,492
	L50	1	1
	N90	1	1
	N’s	0	0
	Avg. coverage depth (Nanopore)	640	545
	Avg. coverage depth (Illumina)	566	687
Assembly (plasmid)	Plasmid length (bp)	1,840	5,865
	Plasmid coverage depth (Nanopore)	760	75
	Plasmid coverage depth (Illumina)	1,233	669
	Plasmid copy number	2.23	0.9
	Plasmid two length (bp)		1,841
	Plasmid 2 coverage depth (Nanopore)		310
	Plasmid 2 coverage depth (Illumina)		901
	Plasmid 2 copy number		1.32
	Plasmid 1 PLSDB hit	–^*a*^	+^*b*^
	Plasmid 2 PLSDB hit	–	–
	NUCCORE_ACC	–	NZ_CP102349.1
BUSCO	*exiguobacterium_odb12*	C: 99.2% [99.2%,D: 0.1%], F: 0.0%, M: 0.8%, *n* = 1779	C: 99.2%[99.2%, D: 0.1%], F: 0.0%, M: 0.8%, *n* = 1779
Annotation (PGAP)	Organism (input)	*Exiguobacterium alkaliphilum*	*E. alkaliphilum*
	Organism (output)	*Exiguobacterium alkaliphilum* (ANI = 98.07%) Status: Confirmed Confidence: High	*E. alkaliphilum* (ANI = 98.07%) Status: Confirmed Confidence: High
	Genes (total)	2,952	2,948
	CDSs (total)	2,853	2,849
	Genes (coding)	2,839	2,835
	CDSs (with protein)	2,839	2,835
	Genes (RNA)	99	99
	rRNAs	10, 9, 9 (5S, 16S, 23S)	10, 9, 9 (5S, 16S, 23S)
	Complete rRNAs	10, 9, 9 (5S, 16S, 23S)	10, 9, 9 (5S, 16S, 23S)
	tRNAs	67	67
	ncRNAs	4	4
	Pseudo genes (total)	14	14
	CDSs (without protein)	14	14
	Pseudo genes (ambiguous residues)	0 of 14	0 of 14
	Pseudo genes (frameshifted)	4 of 14	5 of 14
	Pseudo genes (incomplete)	10 of 14	9 of 14
	Pseudo genes (internal stop)	1 of 14	1 of 14
	Pseudo genes (multiple problems)	1 of 14	1 of 14
	CRISPR arrays	–	–
Annotation (PROKKA)	CDS	2,877	2.88
	Gene	3,018	3,021
	mRNA	3,018	3,021
	misc_RNA	45	45
	rRNAs	28	28
	repeat_region	–	–
	tRNAs	67	67
	tmRNA	1	1
HADEG	A_Alkanes (*AeAB*_*ahpC*)	1	1
	A_Alkanes (*AeAB*_*ahpF*)	1	1
	C_Aromatics (*AeCZGII*_*pchA*)	1	1
	E_Plastics (*AeEP*_*SUBS*_*LEDLE*)	1	1

^*a*^ “–” denotes not detected.

^*b*^ “+” denotes detected.

## Data Availability

BioProject for this study is deposited under PRJNA1274991 number. *E. alkaliphilum* isolates BS282 and BS283 complete genome sequences were deposited in NCBI under accession numbers SRR33987390 and SRR33987391, respectively. Genome assembly was deposited under accession numbers GCA_052497025.1 and GCA_052496835.1.
